# Prevalence and materno-fetal outcomes of preeclampsia/eclampsia amongst pregnant women at a teaching hospital in north-central Nigeria: a retrospective cross-sectional study

**DOI:** 10.1186/s40885-021-00178-y

**Published:** 2021-10-15

**Authors:** Godwin O. Akaba, Ubong I. Anyang, Bissallah A. Ekele

**Affiliations:** 1 Department of Obstetrics and Gynaecology, College of Health Sciences, University of Abuja, Gwagwalada, Nigeria; 2 Department of Obstetrics and Gynaecology, University of Abuja Teaching Hospital, Gwagwalada, Nigeria

**Keywords:** Pre-eclampsia, Eclampsia, Prevalence, Maternal-fetal relations, Risk factors, Maternal mortality, Nigeria

## Abstract

**Background:**

Preeclampsia/eclampsia (PE/E) contributes significantly to maternal, perinatal morbidity and mortality in Nigeria. The objectives of the study were to ascertain the prevalence, materno-fetal outcomes and sociodemographic factors associated with PE/E at Nigerian Teaching Hospital from September 2014 to August 2019.

**Methods:**

This was a retrospective cross-sectional study that analyzed deidentified secondary data of women managed for PE/E at a teaching hospital in north-central, Nigeria. Descriptive statistics were used to determine sample characteristics and study outcome estimates. Bivariate analysis was used to test for associations between sociodemographic factors and PE/E, materno-fetal outcomes while logistic regression analysis was used to test for the magnitude of these associations. The significance level was set at *P* < 0.05.

**Results:**

The prevalence of PE/E in this study was 3.60%. Preeclampsia was diagnosed in 3.02% of cases while eclampsia was the diagnosis in 0.58%. Case fatality rate was 3.9% and still birth rate was 10.7%. Majority of women (85.4%) did not have any maternal complication nor unfavorable outcome. Majority (67.7%), of babies weighed less than 2500 g and birth weight was the only sociodemographic factor that was significantly associated with fetal outcome (X^2^ = 15.6, *P* < 0.001).

**Conclusions:**

The prevalence of PE/E in this study is high and is associated with high maternal and perinatal deaths. Majority of the cases of PE/E as well the fatalities occurred in women who had no formal education, unbooked and referred to the teaching hospital with worsening conditions. There is need for explorative research on community factors associated with PE/E and its outcome towards prevention and early management of cases.

## Background

Preeclampsia/eclampsia (PE/E) is a major public health problem globally and in Nigeria [1]. Globally, PE/E complicate up to 4.6 and 1.4% of pregnancies respectively but disproportionately accounts for nearly 18% of all maternal deaths worldwide, with an estimated 62,000 to 77,000 deaths per year [2]. Perinatal health is also negatively affected as an estimated 500,000 babies die each year from PE/E [3].

Regional differences exist in the prevalence and mortality associated with PE/E. Women in low-resource countries are at a higher risk of developing preeclampsia compared with those in high-resource countries [3]. They are also at increased risk of maternal and perinatal morbidities and mortalities from these conditions due to lack of prenatal care, lack of access to hospital care, lack of resources, and inappropriate diagnosis and management of patients with PE/E in the developing countries [4].

Relative to other low-income, sub-Saharan African nations, Nigeria has high maternal mortality ratio (512 per 100,000 live births), high fertility rate (5.3 children per woman), and high infant mortality rate (67 deaths per 1000 live births) [5].

PE/E is a leading cause of maternal and perinatal mortality in Nigeria and also associated with adverse maternal and fetal outcomes [6, 7]. A Nationwide cross-sectional study of 998 maternal deaths and 1451 near misses in public tertiary hospitals in Nigeria showed that PE/E was the highest contributor to maternal deaths being the cause of maternal deaths in 28.3% of cases [8]. Eclampsia alone contributed to 42.2% of maternal deaths in Sokoto, Northern Nigeria [9].

Despite its overwhelming contribution to maternal and perinatal morbidity and mortality, the prevalence, maternal and fetal outcomes of patients managed with PE/E are yet to be fully evaluated in Nigeria’s Federal Capital Territory, Abuja. A previous retrospective cross-sectional study from the University of Abuja Teaching Hospital analyzed only data of women with eclampsia from 2005 to 2008 and did not evaluate for risk factors associated with materno-fetal outcomes neither did it report on preeclampsia which is an important precursor of eclampsia [10].

The present study was designed to determine the prevalence of PE/E as well as compare outcomes between preeclampsia and eclampsia and test for associations between sociodemographic risk factors and materno-fetal outcomes. Findings from this study will provide baseline data on the recent prevalence of PE/E in Nigeria’s Federal Capital since after the launch of the Sustainable Development Goals and help determine the outcome of women with PE/E and by extension their health condition at presentation and the quality of care the women receive at the teaching hospital. The finding may help address necessary gaps in patients care and help in health care planning and improved service delivery in Abuja.

## Methods

### Study design

This is a retrospective cross-sectional study which used information extracted from deidentified patients’ medical records using a proforma.

### Study location/setting

The study was conducted at the Department of Obstetrics and Gynaecology of University of Abuja Teaching Hospital, Gwagwalada. The hospital is a 350-bed federal government-owned tertiary institution situated in Gwagwalada; a semi-urban town located in the Gbagyi speaking region of the Federal Capital Territory (FCT). It provides health care services to the inhabitants of the Nation’s FCT and neighboring states in north-central Nigeria (Niger, Kaduna, Kogi and Nasarawa). The Department of Obstetrics and Gynaecology undertakes about 2000 to 2500 deliveries annually.

### Sampling frame

Medical records of all women managed for PE/E between September 2014 and August 2019 were retrieved, and variables related to sociodemographic characteristics, maternal and fetal outcomes were extracted using a proforma designed specifically for the study. Additionally, the total number of deliveries occurring during the period was obtained towards ascertaining the prevalence of PE/E.

### Sample size calculation

The sample size for the study was calculated using the formula for calculating sample size for cross-sectional studies [11].

*n =* Z^2^p (1 – p)/d^2^ where,

*n =* minimum sample size, Z = standard normal variate (at 5% type 1 error, *P* < 0.05) = 1.96.

*p* = prevalence of 8.8% for preeclampsia based on a previous study in Jos, north-central Nigeria [12].

*N* = 1.96^2^ × 0.088 (1–0.0088) = 123.3.

0.05^2^

Considering an attrition of 15% attributable to failure of retrieval of case folders, the total sample size was be approximated to 142. Overall, 335 cases were reviewed for analysis.

### Inclusion criteria

All pregnant women presenting to the Obstetrics and Gynaecology unit of the University of Abuja Teaching Hospital during the study period and managed for PE/E were included into the study.

### Exclusion criteria

The exclusion criteria were specified as follows: (1) Women who delivered before 1st of September, 2014 and after 30th of August, 2019; (2) Women who were diagnosed to have chronic hypertension before onset of pregnancy or before 20th week of pregnancy; (3) Women who did not have PE/E.

### Data collection and data analysis

Anonymized data was extracted unto a proforma and the information on the proformas were entered into a computer. Data analysis was performed using IBM SPSS ver. 20.0 (IBM Corp., Armonk, NY, USA). Descriptive analysis of rates of PE/E, frequencies of all variables were done.

The proportion of pregnant women with PE/E to the total number of deliveries during the study period was used to calculate the prevalence while medical records with documentation of the variables of interest (age, parity, estimated gestational age, booking status, antenatal clinic attendance, maternal outcomes, fetal outcomes) were used to assess maternal and fetal outcomes. The main primary maternal outcome was maternal death while the main primary fetal outcome was stillbirth. Mean age of the patient, parity, gestational age at delivery, fetal birth weight, and APGAR (Appearance, Pulse, Grimace, Activity, and Respiration) scores at the first and fifth minutes of birth were calculated. Chi-squared test was used to ascertain the association between independent variables (age, parity, educational status, booking status, and past history of PE/E) and maternal deaths and stillbirths. Logistic regression analysis was used subsequently to identify factors which appear to be independently associated with maternal deaths or stillbirths.

Additional ward and theatre registers were reviewed to identify any data that was missing in the case notes. These were complimented by the use of nurse’s inpatient treatment notes and registers. Cases without documentation of the main primary maternal and fetal outcomes were to be excluded from the analysis but none was in this category.

### Operational definitions of outcome variables

Maternal complications or unfavorable maternal outcomes refers to mothers who had at least one of the following complications: eclampsia, acute renal failure, stroke, intracranial hemorrhage, disseminated intravascular coagulation, HELLP (hemolysis, elevated liver enzymes, low platelets) syndrome, cardiac failure, abruptio placenta, aspiration pneumonia, or pulmonary edema.

Fetal complications or unfavorable fetal outcome refers to newborns who had at least one of the following complications: preterm delivery, low birth weight (LBW), admission to the neonatal intensive care unit, low APGAR score (Apgar score less than 7 in either or both 1st and 5th min of delivery).

Stillbirth was defined as death of a fetus weighing at least 1000 g or 28 completed weeks of gestation occurring at the complete expulsion or extraction from its mother in accordance with the World Health Organization (WHO)‘s agreed definition of stillbirth for international comparison [13].

## Results

### Prevalence of PE/E

During the 5-year period under review, the hospital recorded a total of 9760 deliveries of which 352 were women managed for PE/E thereby giving a total prevalence of 3.60%. Preeclampsia was diagnosed in 295 of cases (3.02%) while eclampsia was the diagnosis in 57 (0.58%).

**Fig. 1:**
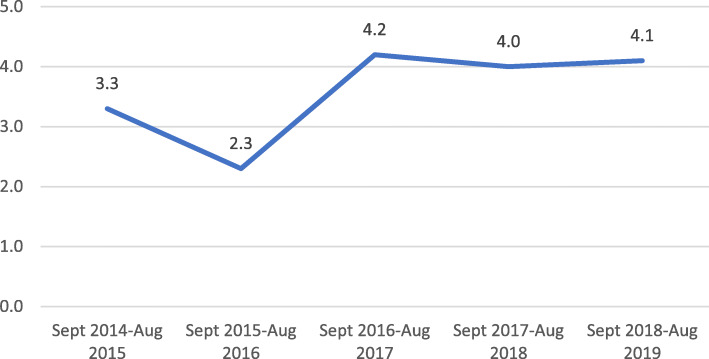
Yearly trend in the prevalence of preeclampsia/eclampsia during the study period

Figure 1 shows the yearly trend in the prevalence of PE/E during the study period**.** The yearly prevalence of PE/E ranged from 2.3 to 4.2% with a peak in the period of September 2016 to August 2017, the later years were quite consistent at approximately 4.0%.

### Sociodemographic and clinical characteristics of patients with PE/E

Out of the 352 cases of PE/E managed, 335 patients’ case notes (95.17%) had adequate information and were used for analysis of maternal and fetal outcomes. The age of the participants ranged from 15 to 45 years with a mean of 30.1 ± 5.87. The majority of the participants were married 322 (96.1%), had only secondary or tertiary education 262 (78.2%) and were nulliparous at admission in labour ward, 122 (36.4). Regarding antenatal care attendance, majority, 306 (91.3%) booked and attended ante natal care (ANC) either at a private clinic/maternity center while only 29 (8.7%) did not attend ANC. This is shown in Table 1.

**Table 1:** Characteristics and clinical features of the participants

Characteristic	Preeclampsia	Eclampsia	PE/E (*n* = 335)
Age (yr)			
≤ 19	5 (1.8)	9 (16.7)	14 (4.2)
20–24	28 (10.0)	13 (24.1)	41 (12.2)
25–29	80 (28.5)	12 (22.2)	92 (27.5)
30–34	91 (32.4)	14 (25.9)	105 (31.3)
≥ 35	77 (27.4)	6 (11.1)	83 (24.8)
Mean	30.8	26.7	30.1
Median (interquartile range)	31.0 (27–35)	26.5 (21–30)	30.0 (26–34)
Marital status			
Single	2 (0.8)	3 (5.6)	5 (1.5)
Married	271 (96.4)	51 (94.4)	322 (96.1)
Divorced	8 (2.8)	0	8 (2.4)
Types of education			
No formal education	6 (2.1)	7 (13.0)	13 (3.9)
Primary	38 (13.5)	11 (20.4)	49 (14.6)
Secondary	120 (42.7)	26 (48.1)	146 (43.6)
Tertiary	108 (38.4)	8 (14.8)	116 (34.6)
Missing	9 (3.3)	2 (3.7)	11 (3.3)
Parity			
Nulliparous	97 (34.5)	25 (46.3)	122 (36.3)
Primiparous	60 (21.4)	12 (22.2)	72 (21.5)
Multiparous	93 (33.1)	12 (22.2)	105 (31.3)
Grand multiparous	31 (11.0)	5 (9.3)	36 (10.7)
Booking status			
Unbooked	15 (5.3)	14 (25.9)	29 (8.7)
Booked at the teaching hospital	91 (32.4)	3 (5.6)	94 (28.1)
Booked elsewhere at PHC	53 (18.9)	17 (31.5)	70 (20.9)
Booked in a secondary health center	97 (34.5)	14 (25.9)	111 (33.1)
Booked in a private clinic/maternity center	25 (8.9)	6 (11.1)	31 (9.3)
Attend ANC			
No	15 (5.3)	14 (25.9)	29 (8.7)
Yes	266 (94.7)	40 (74.1)	306 (91.3)
Place of ANC			
Teaching hospital	84 (29.9)	2 (3.7)	86 (25.7)
General hospital	107 (38.1)	15 (27.8)	122 (36.4)
Primary health care	48 (17.1)	15 (27.85)	63 (18.8)
Private clinic/maternity home	27 (9.6)	8 (14.8)	35 (10.4)
No ANC attended	15 (5.3)	14 (25.9)	29 (8.7)

Data are presented as number (%) unless otherwise specified

*PE/E* preeclampsia/eclampsia, *PHC* primary healthcare center, *ANC* ante natal care

### Bivariate and multivariate analysis for factors associated with eclampsia

When women with PE/E outcomes were compared, robust significant relationships were found among eclampsia and women within ages of less than 24 years X^2^ = 29.3, *P* < 0.001, women with no formal education X^2^ = 22.8, *P <* 0.001, unbooked women at delivery X^2^ = 38.4, *P <* 0.001, women who did not attend ANC X^2^ = 21.7, *P* < 0.001.

Following multivariate analysis, women who were aged 25 to 29 years had an 80% decrease in the odds of being diagnosed with eclampsia when compared with women less than 24 years. Primary, secondary and tertiary education were protective for eclampsia. Furthermore, multiparous women were less likely to be diagnosed with eclampsia with a 50% decrease in odds. Also, women who attended ANC were less likely with a 90% decrease in the odds of being diagnosed with eclampsia than women who did not attend ANC. The results are presented in Table 2 below.

**Table 2:** Odd ratios from logistic regression for factors associated with eclampsia

Variable	Adjusted odd ratio	95% confidence interval	*P*-value
Age group (yr)			
≤ 24 (RC)	1		
25–29	0.225	0.100–0.507	0.037*
30–34	0.231	0.106–0.503	0.001*
≥ 35	0.117	0.117–0.315	0.001*
Marital status			
Married (RC)	1		
Not married	1.594	0.424–5.994	0.490
Type of education			
No formal education (RC)	1		
Primary	0.248	0.069–0.893	0.033*
Secondary	0.186	0.058–0.598	0.005*
Tertiary	0.063	0.017–0.234	0.001*
Booking status			
Unbooked (RC)	1		
Booked at the teaching hospital	0.035	0.009–0.138	0.001*
Booked elsewhere at PHC	0.344	0.138–0.854	0.021*
Booked in a secondary health center	0.155	0.062–0.388	0.000*
Booked in a private clinic/maternity center	0.257	0.081–0.813	0.021*
Parity			
Nulliparous (RC)	1		
Primiparous	0.776	0.363–1.659	0.513
Multiparous	0.501	0.238–1.054	0.069
Grand multiparous	0.626	0.221–1.774	0.378
Attend ANC			
No (RC)	1		
Yes	0.161	0.072–0.359	0.001*
Place of ANC			
Teaching hospital	0.026	0.005–0.124	0.001*
General hospital	0.150	0.061–0.372	0.001*
Primary health care1	0.335	0.132–0.849	0.021*
Private clinic/maternity home	0.317	0.108–0.929	0.036*
No ANC attended (RC)	1		

*RC* reference category, *PHC* primary healthcare center, *ANC* ante natal care

*P < 0.05

### Maternal outcomes

Of the 335 women with PE/E, 281 (83.9%) had preeclampsia at delivery while 54 (16.1%) had eclampsia. Overall, 286 of the cases (85.4%) did not have any maternal complication nor unfavorable outcome. Majority of the women, 322 (96.1%) were discharged home alive while 13 (3.9%) were maternal deaths. These are as shown in Table 3.

**Table 3:** Maternal outcomes of preeclampsia/eclampsia

Characteristic	Preeclampsia	Eclampsia	PE/E (*n* = 335)
Maternal complications			
No	249 (88.6)	37 (68.5)	286 (85.4)
Yes	32 (11.4)	17 (31.5)	49 (14.6)
Primary maternal outcome			
Maternal death	10 (3.6)	3 (5.6)	13 (3.9)
Alive	271 (96.4)	51 (94.4)	322 (96.1)
Route of delivery			
Vaginal delivery	50 (17.8)	17 (31.5)	67 (20.0)
Instrumental vaginal	17 (6.0)	3 (5.6)	20 (6.0)
Caesarean section	214 (76.2)	34 (63.0)	248 (74.0)

Data are presented as number (%)

### Bivariate analysis for factors associated with primary and secondary maternal outcomes

None of the factors tested (age, marital status, educational level, booking status, parity, ANC attendance and place of ANC) were found be significantly associated with primary maternal outcomes. Similar and comparable to primary maternal outcomes, factors such age, marital status, educational level, booking status, parity and place of ANC were found not be significantly associated with secondary maternal outcomes.

### Fetal outcomes

Amongst the 319 whose babies birth weights were recorded, majority, 216 (67.7%) weighed less than 2500 g. A total of 190 (56.7%) had good apgar scores of ≥7 in the first minute of birth. This number increased to 246 (73.4%) in the fifth minute of birth following neonatal resuscitation. Unfortunately, 10 babies (3.0%) who had apar scores of 1 to 3 in the first minute of life suffered immediate perinatal deaths. Overall, 299 of the babies (89.3%) were born alive while 36 of the babies (10.7%) were stillbirths. These are as shown in Table 4.

**Table 4:** Fetal outcomes of preeclampsia/eclampsia

Characteristic	Preeclampsia	Eclampsia	PE/E (*n* = 335)
Fetal outcome			
Stillbirth	30 (10.7)	6 (11.1)	36 (10.7)
Alive	251 (89.3)	48 (88.9)	299 (89.3)
APGAR scores 1st minute			
0	27 (9.6)	9 (16.7)	36 (10.7)
1–3	25 (8.9)	8 (14.8)	33 (9.9)
4–6	62 (22.1)	14 (25.9)	76 (22.7)
≥ 7 and above	167 (59.4)	23 (42.6)	190 (56.7)
APGAR scores 5th minute			
0	35 (12.5)	11 (20.4)	46 (13.7)
1–3	9 (3.2)	3 (5.6)	12 (3.6)
4–6	26 (9.3)	5 (9.3)	31 (9.3)
≥ 7	211 (75.1)	35 (64.8)	246 (73.4)
Birth weight (g)			
< 1000	6 (2.2)	2 (4.3)	8 (2.5)
1000 to < 1500	59 (21.5)	4 (13.0)	63 (19.7)
1500 to < 2500	126 (45.8)	19 (41.3)	145 (45.5)
2500 to < 4000	74 (26.9)	18 (39.1)	92 (28.8)
≥ 4000	10 (3.6)	1 (2.2)	11 (3.5)

Data are presented as number (%)

*APGAR* Appearance, Pulse, Grimace, Activity, and Respiration

### Bivariate analysis for factors associated with primary fetal outcome

Amongst all the factors tested, fetal birth weight was found to be statistically significantly associated with whether the babies were born alive or dead, X^2^ (4, *N* = 315) = 15.6, *P* < 0.001. Factors such age, marital status, educational level, booking status, parity, ANC attendance and place of ANC were found not be significantly associated with primary fetal outcome. Results are displayed in Table 5.

**Table 5:** Bivariate analysis for factors associated with primary fetal outcome

Variable	Fetal outcome		^Χ2^, (df)	*P-*value
	Stillbirth	Alive		
Age group (yr)				
≤ 24	2 (5.6)	53 (17.7)	4.505, (3)	0.234
25–29	9 (25.0)	83 (27.8)		
30–34	14 (38.9)	91 (30.4)		
≥ 35	11 (30.6)	72 (24.1)		
Marital status				
Not married	1 (2.8)	12 (4.0)		1.000^a)^
Married	35 (97.2)	287 (96.0)		
Type of education				
No formal education	1 (2.8)	12 (4.2)	3.178, (3)	0.365
Primary	9 (25.0)	40 (13.9)		
Secondary	14 (38.9)	132 (45.8)		
Tertiary	12 (33.3)	104 (36.1)		
Booking status				
Unbooked	5 (13.9)	24 (8.0)	3.617, (4)	0.460
Booked at the teaching hospital	6 (16.7)	88 (29.4)		
Booked elsewhere at PHC	9 (25.0)	61 (20.4)		
Booked in a secondary health center	12 (33.3)	99 (33.1)		
Booked in a private clinic/maternity center	4 (11.1)	27 (9.0)		
Parity				
Nulliparous	13 (36.1)	109 (36.5)	2.453, (3)	0.484
Primiparous	5 (13.9)	67 (22.4)		
Multiparous	12 (33.3)	93 (31.1)		
Grand multiparous	6 (16.7)	30 (10.0)		
Attend ANC				
No	5 (13.9)	24 (8.0)		0.219^a)^
Yes	31 (86.1)	275 (92.0)		
Place of ANC				
Teaching hospital	6 (16.7)	80 (26.8)	2.818, (4)	0.589
General hospital	13 (36.1)	109 (36.5)		
Primary health care	8 (22.2)	55 (18.4)		
Private clinic/maternity home	4 (11.1)	31 (10.4)		
No ANC attended	5 (13.9)	24 (8.0)		
Birth weight (g)				
< 1000	2 (6.1)	6 (2.1)	17.377, (4)	0.002*
1000 to < 1500	12 (36.4)	53 (18.4)		
1500 to < 2500	17 (51.5)	128 (44.4)		
2500 to < 4000	2 (6.1)	90 (31.2)		
≥ 4000	0	11 (3.8)		
Diagnosis at delivery				
Preeclampsia	30 (83.3)	251 (83.9)	0.009, (1)	0.925
Eclampsia	3 (23.1)	51 (15.8)		

Data are presented as number (%) unless otherwise indicated

^Χ2^, chi-square value; *df* degree of freedom, *PHC* primary healthcare center, *ANC* ante natal care

^a)^ Fishers exact test

*P-value < 0.05 is significant

## Discussions

The study showed a prevalence of 3.6% for PE/E, with PE/E contributing 3.02 and 0.58% respectively to the overall prevalence of PE/E in the institution. This finding is comparable to a prevalence of 3.12% for PE/E reported for Nigeria from 2006 to 2008 in a facility-based secondary analysis of the WHO Global Survey on Maternal and Perinatal Health involving 24 countries including eight African countries [14].

When compared with another facility-based data from Nigeria, the prevalence rate of PE/E found in this study was similar to 4.0% (preeclampsia, 3.4%; eclampsia, 0.6%) reported by researchers in Abakaliki, Southeast Nigeria [15]. The similar findings may be due to the fact that both studies were conducted in Federal Teaching Hospitals that receive referrals from primary, secondary and private hospitals from within their states and neighboring states. Additionally, the sociodemographic variables like age and parity were similar.

The prevalence of preeclampsia alone from this study is similar to 3.53% quoted in a retrospective cross-sectional study from Bayelsa, South South, Nigeria [16] but lower than 6.0% reported respectively by Singh et al. [17], in a prospective cohort study in Sokoto, Northwest, Nigeria. The higher prevalence rate in the later study may be due to the small sample size of the study which was composed of 216 pregnant women out of which 10 developed preeclampsia.

Regarding prevalence of eclampsia, findings from this current study is comparable to 0.6% reported by Onoh et al. [15]. Other studies in Nigeria have however reported higher prevalence of eclampsia of 0.91 to 9.42% [6, 16, 18–22]. The high prevalence seen in these referenced studies may be due to several factors including high percentage of women who were unbooked and therefore did not receive ANC services in these hospitals. Our study findings support the later assumption as women who did not attend ANC had higher odds of developing eclampsia. WHO currently recommends that pregnant women should have at least 8 ANC contacts towards a positive pregnancy experience [23]. Unfortunately, in Nigeria only 67% of women receive ANC from a skilled provider during the pregnancy with just 57% having at least four ANC visits [5]. Since the cause of PE/E remains hugely poorly understood, screening and closer monitoring of women with these risk factors may help in prevention, early detection and treatment. This is even much more important at the primary health care centers, general hospitals and private hospitals where majority of these women register and receive ANC.

The prevalence rate and case fatality from eclampsia decreased respectively from 1.3 to 0.58% and 8.5 to 3.0% when compared with findings from the same institutions about a decade ago [10]. This reduction in prevalence and case fatality could reflect improved management of cases of preeclampsia in the institution because of training of health workers and protocol development on management of preeclampsia.

Unfavorable maternal outcomes from PE/E like abruptio placentae, HELLP syndrome, pulmonary oedema and renal failure are well documented [3, 24]. Any of these or their combination was seen in 49 of cases (14.6%) and is comparable to findings by Onoh et al. [15], where 46/254 (18.1%) developed these complications.

Caesarean delivery rates in studies that reported routes of delivery in women with PE/E in Nigeria have ranged between 48.42 to 71.2% [15, 16, 22, 25]. The attributing factor to the high rate of caesarean section in this study was because majority of the women were referred late to the hospital with other complications of PE/E or obstetric indications requiring caesarean section for optimal maternal and fetal outcome.

The still birth rate reported in this study although comparatively lower than previous reports from Nigeria of 12.3% [26], 29.1% [15], and 36.84% [16]. highlights the huge contribution of PE/E to the high burden of perinatal mortality in Nigeria. When compared to reports by Onoh et al. [15], the percentage of babies that had good APGAR scores in the first and fifth minutes of life in this study was similar (current study: 56.7% vs. 57.2%) and (current study: 73.4% vs. 72.8%), respectively. This suggests ability of the obstetric units in Nigeria to salvage babies with mild birth asphyxia but not particularly so for severely asphyxiated babies in which some suffered immediate perinatal deaths.

This study also revealed the significant contribution of PE/E to the burden of prematurity and LBW babies in Nigeria with their attendant complications of stillbirths/early perinatal deaths. Well documented and effective interventions for managing preterm babies includes the use of antenatal corticosteroids, continuous positive airways pressure ventilation, exogenous surfactant administration, Kangaroo mother care, prevention of hypothermia using plastic bag/wrap and cap, early initiation of breast milk feeding [27, 28]. These interventions should be vigorously implemented in all obstetric units in Nigeria. Additionally, training and retraining of health care workers at referring health facilities on how to identify fetuses at risk of prematurity/LBW for prompt referral to higher levels of care, availability of human and material resources for neonatal resuscitation would be useful towards improving fetal outcomes.

Factors such as age, marital status, educational level, booking status, parity, ANC attendance and place of ANC were found not to be significantly associated with primary maternal and fetal outcomes. This is similar to findings from a retrospective review of maternal deaths from eclampsia by Nwafor [29] in which all sociodemographic variables did not show any statistically significant association with maternal outcome. This suggests that apart from addressing sociodemographic variables which are risk factors of PE/E, there is dire need to identify and address community and hospital-based factors contributing to maternal deaths from PE/E. These includes increased health promotion campaigns on recognition of danger signs of pregnancy, improved education/awareness, discouragement of cultural practices and negative beliefs regarding PE/E, encouragement to attend ANC, need to present to hospital early, prompt treatment/stabilization and early referral to higher centers by health care providers, and improved quality of care in the hospitals.

The retrospective cross-sectional design of the study may make it difficult to derive causal relationships and limits the generalizability of the results. It therefore makes case for a prospective coordinated PE/E registry to monitor maternal-fetal outcomes in Nigerian obstetric populations.

## Conclusions

The prevalence of PE/E in this study is high and associated with high maternal and perinatal deaths. Majority of the cases of PE/E as well the fatalities occurred in women who had no formal education, did not attend ANC and referred to the teaching hospital with worsening conditions. Sociodemographic factors did not seem to affect maternal outcomes, but fetal birthweight was associated with still births.

Efforts to address the problem of PE/E and the poor maternal and fetal outcomes associated with the condition must be put in place for Nigeria to meet Sustainable Development Goals 3. This will involve strengthening of health systems at all levels of health care provision in the FCT and Nigeria in general as well as participation of several stakeholders like community leaders, religious leaders, non-governmental organizations, Health Ministry and Education Ministry.

## Data Availability

The data sets generated during and/or analyzed during the current study are available from the corresponding author on reasonable request.

## Abbreviations

ANC: Ante natal care
APGAR: Appearance, Pulse, Grimace, Activity, and Respiration
HELLP: Haemolysis, elevated liver enzymes, low platelets
LBW: Low birth weight
PE/E: Preeclampsia/eclampsia
PHC: Primary healthcare center
RC: Reference category
WHO: World Health Organization
